# Age-Dependent Retinal Parameter Correlation Patterns on OCT and OCT Angiography in Children and Adults

**DOI:** 10.3390/jcm15124778

**Published:** 2026-06-19

**Authors:** Claudia Lommatzsch, Antoine Capucci, Swaantje Grisanti, Carsten Heinz, Kai Rothaus

**Affiliations:** 1 Department of Ophthalmology at St. Franziskus-Hospital Muenster, Hohenzollernring 74, 48145 Muenster, Germany; antoine.capucci@augen-franziskus.de (A.C.); carsten.heinz@augen-franziskus.de (C.H.); kai.rothaus@augen-franziskus.de (K.R.); 2 Department of Ophthalmology, University of Luebeck, 23538 Luebeck, Germany; swaantje.grisanti@uksh.de; 3 Department of Ophthalmology, University of Essen, Hufelandstrasse 55, 45147 Essen, Germany

**Keywords:** optical coherence tomography, OCT angiography, pediatric ophthalmology, correlation analysis, retinal development, normative data

## Abstract

**Background/Objectives**: Optical coherence tomography (OCT) and OCT angiography (OCT-A) provide detailed measurements of retinal structure and vasculature; however, age-related differences in how these parameters correlate with one another remain poorly understood. We hypothesized that vascular–structural integration in the macula is more pronounced in adults than in children. Our aim was to characterize correlation patterns in pediatric and adult populations to inform the development of age-specific clinical interpretation guidelines. **Methods**: This prospective cross-sectional observational study enrolled 37 healthy children (age 1–17 years) and 28 healthy adults (age 18–65 years). Eyes with ocular or systemic conditions affecting the retina or prior intraocular surgery were excluded. Standardized OCT and OCT-A acquisition protocols provided structural and vascular measures. Univariable correlation analyses applied a stringent threshold (*p* < 0.001) to identify robust associations. Significant univariable results were entered into multivariable regression models adjusting for age, gender, intraocular pressure, and axial length. A Group-wise Linkage Proportion quantified the percentage of potential significant correlations among eight predefined anatomical parameter groups. **Results**: Ninety univariable correlations met *p* < 0.001. Fourteen correlations were shared across age groups, notably foveal avascular zone metrics and vessel density, showing very large negative correlations (r = −0.70 to −0.87). The pediatric cohort displayed 40 unique correlations, primarily linking optic nerve head flow indices to retinal nerve fiber layer thickness. Adults exhibited 36 unique correlations, dominated by macular vascular–thickness coupling concentrated in the parafoveal region. After multivariable adjustment, 52 of 90 associations remained significant. Adult-specific associations lost significance more frequently (58%) than pediatric-specific associations (43%), whereas correlations shared across both groups showed complete stability (100%). The Group-wise Linkage Proportion indicated pronounced macular vascular–structural coupling in adults (48.4%) versus near absence in children (1.2%). **Conclusions**: Retinal parameter correlation patterns show fundamental differences between pediatric and adult eyes. While optic nerve head-macular thickness relationships remain consistent across ages, adults exhibit mature, localized integration of macular vascular and structural parameters absent in children. These findings suggest that pediatric and adult OCT/OCT-A measurements may benefit from separate reference standards, although prospective validation is required before clinical implementation.

## 1. Introduction

The human retina is a complex neurovascular tissue in which structural architecture and microvascular perfusion are closely interrelated. Optical coherence tomography (OCT) and OCT angiography (OCT-A) provide complementary, high-resolution measurements of retinal layer architecture and capillary networks, enabling simultaneous characterization of structural and perfusion parameters that are interdependent and clinically informative. Recent advances in optical imaging technology, including improved image processing algorithms and enhanced optical components, continue to refine image quality and resolution in OCT-based systems [1,2]. Normative values for key OCT and OCT-A metrics have been reported for both adult and pediatric populations, and correlations among retinal structural and vascular parameters have been characterised within age-homogeneous cohorts. Accurate interpretation of multimodal retinal imaging therefore requires not only knowledge of individual normative values but also an understanding of the typical interparameter relationships that reflect underlying anatomy and physiology.

Although numerous studies have described age-related trajectories of single OCT or OCT-A metrics and have characterized correlation patterns within age-homogeneous pediatric or adult cohorts, direct comparative analyses of correlation structures across childhood and adulthood remain scarce. To our knowledge, no prior study has directly compared inter-parameter correlation structures between childhood and adulthood using a standardised multimodal protocol. This gap has direct clinical consequences, as correlation patterns established in adult cohorts are routinely applied to guide the interpretation of pediatric imaging findings. The retina continues to undergo structural and vascular maturation into late adolescence [3,4], and these developmental changes may alter how retinal parameters relate to each other. This knowledge gap makes pediatric imaging interpretation difficult: applying adult reference patterns to children may lead to misclassification of normal developmental findings as pathological, while potentially overlooking true pathological changes that alter the relationship between structural and vascular measurements. Furthermore, emerging computational and machine learning approaches to retinal image analysis have highlighted the multidimensional nature of structure–perfusion relationships, underscoring the need for age-appropriate inter-parameter reference frameworks as a necessary foundation for such analyses. To address this gap, we performed a comprehensive cross-sectional correlation analysis of OCT and OCT-A parameters in healthy children and adults using standardized acquisition and statistical protocols. Our aims were to identify age-specific correlation patterns in different retinal regions, to test whether these correlations remain significant after adjusting for confounding factors (age, gender, intraocular pressure, axial length), and to describe differences in correlation patterns that can guide the development of age-appropriate reference standards.

## 2. Materials and Methods

### 2.1. Study Design and Participants

This prospective cross-sectional single-center study was performed at the Department of Ophthalmology, St. Franziskus-Hospital Münster, between 1 June 2019 and 1 August 2025, with ethical approval from the Ethics Committee of the Medical Association of Westfalen-Lippe (No. 2019-245-f-s). Informed written consent was obtained from the parents or legal guardians of all participating minors. Consecutive healthy Caucasian volunteers were recruited to represent the range of patients encountered in routine clinical practice across pediatric and adult age groups. Exclusion criteria included inadequate cooperation precluding reliable imaging, media opacities or any fundus-obscuring condition affecting image quality, documented retinal vascular or macular disease, cardiovascular disease or current vasoactive medication, prior intraocular surgery, and history of premature birth. One eye per subject was analysed with a predefined preference for the right eye when both eyes met the inclusion criteria. Prespecified pediatric age strata were 1–9 and 10–17 years; adult strata were 18–31 and ≥32 years.

### 2.2. Clinical Assessment

Participants underwent a standardized ophthalmic examination including subjective refraction and best-corrected visual acuity (BCVA), slit-lamp inspection of the anterior segment, binocular indirect ophthalmoscopy of the posterior segment, and intraocular pressure (IOP) measurement with Icare rebound tonometer; ocular biometry (axial length (AL), lens thickness, and anterior chamber depth), pupil diameter was obtained using the IOL-Master 700 (Carl Zeiss Meditec AG, Jena, Germany).

### 2.3. Retinal Imaging Protocols

Retinal imaging was performed on the Zeiss CIRRUS HD-OCT with AngioPlex technology (spectral-domain OCT, software v.11; Carl Zeiss Meditec AG, Jena, Germany), operating at a scan rate of 68,000 A-scans per second with an axial resolution of approximately 5 µm in tissue. The OCT-A module employs the Optical Microangiography (OMAG) algorithm for flow detection, and motion correction was performed automatically via the integrated FastTrac™ retinal tracking system.

All scans were acquired under non-dilated pupils to avoid mydriatic-induced hemodynamic changes. Image quality thresholds were enforced (signal strength ≥ 6 for structural OCT, ≥8 for OCT-A). Full technical details of the acquisition protocols, segmentation definitions, and parameter specifications have been reported previously using the same cohort and identical imaging setup [5]. All pediatric scans achieved excellent image quality, with no exclusions due to inadequate signal strength or motion artifacts. This demonstrates the technical feasibility of obtaining reliable OCT and OCT-A measurements in pediatric populations when standardized protocols are employed. The Zeiss CIRRUS AngioPlex system employs integrated FastTrac™ retinal tracking for real-time motion correction; scans with residual motion or blink artifacts would have been excluded, but no such exclusions were necessary. The Zeiss CIRRUS system does not apply automatic lateral magnification correction; to account for potential magnification-related effects, axial length was included as a covariate in all multivariable regression models.

### 2.4. Structural OCT Assessment

1. **Peripapillary analysis:** Retinal nerve fiber layer (RNFL) thickness quantification via Optic Disc Cube 200 × 200 protocol, providing quadrant-based and clock-hour sectoral measurements.
2. **Macular structural assessment:** Automated segmentation from inner limiting membrane (ILM) to retinal pigment epithelium (RPE) using Macular Cube 512 × 128 protocol, generating thickness maps across central, parafoveal (3 mm), and perifoveal (6 mm) regions with quadrant subdivision.
3. **Ganglion cell–inner plexiform layer (GCIPL) analysis:** Combined ganglion cell layer (GCL) and inner plexiform layer (IPL) thickness measurement within a 6 × 6 mm acquisition area, displayed as an elliptical annulus with six-segment analysis.

### 2.5. Vascular Assessment

**Figure 1:**
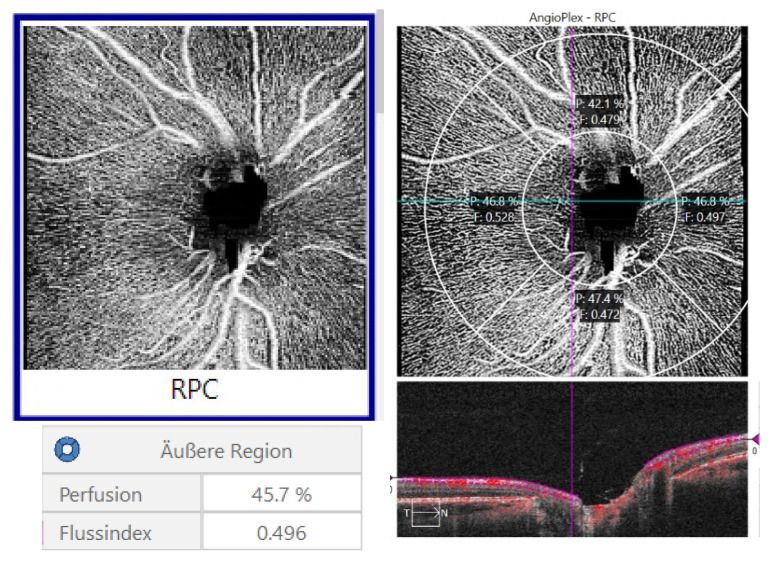
AngioPlex OCT-A: ONH (optic nerve head) angiography 4.5 × 4.5 mm^2^. The en face image shows radial peripapillary capillaries (RPC) with ETDRS-based sector analysis. Quantitative assessment includes *Perfusion (perfusion (PF))* and *Flussindex (flow index (FI))* values displayed for the *Äußere Region (outer region)*. Measurements are presented within the circular analysis grid centered on the optic nerve head.

**Figure 2:**
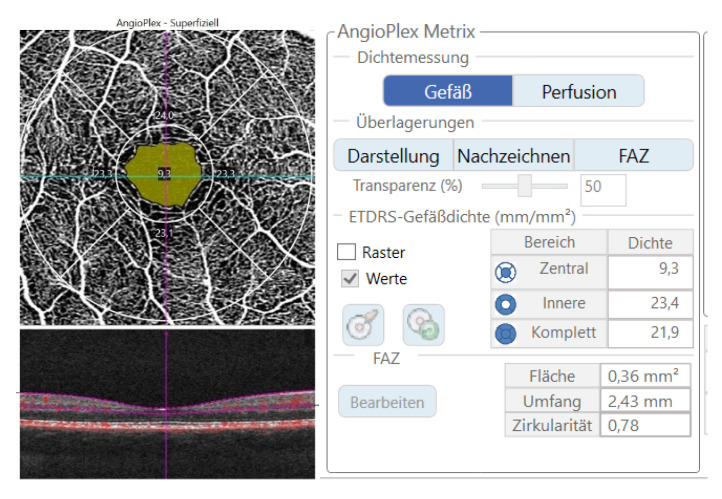
Macular OCT-A 3 × 3 mm^2^ scan analyzed using AngioPlex Metrix. The software interface includes *Gefäß (vessel)* and *Perfusion (perfusion)* display modes with optional *Überlagerungen (overlays)* and adjustable *Transparenz (transparency, %)*. Quantitative *Dichtemessung (density measurement)* comprises *ETDRS-Gefäßdichte (ETDRS vessel density (VD))* and *ETDRS-Perfusionsdichte (ETDRS perfusion density (PD))*. Results are presented by *Bereich (region)* as *Zentral (central)*, *Innen (inner)* and *Komplett (complete)*, with corresponding *Dichte (density)* values. Additional options include *Raster (grid)* and *Werte (values)* display, as well as *Nachzeichnen (tracing)* and *Bearbeiten (edit)* functions. The *FAZ (foveal avascular zone)* analysis provides *Fläche (area)*, *Umfang (perimeter)*, and *Zirkularität (circularity)* measurements.

**Figure 3:**
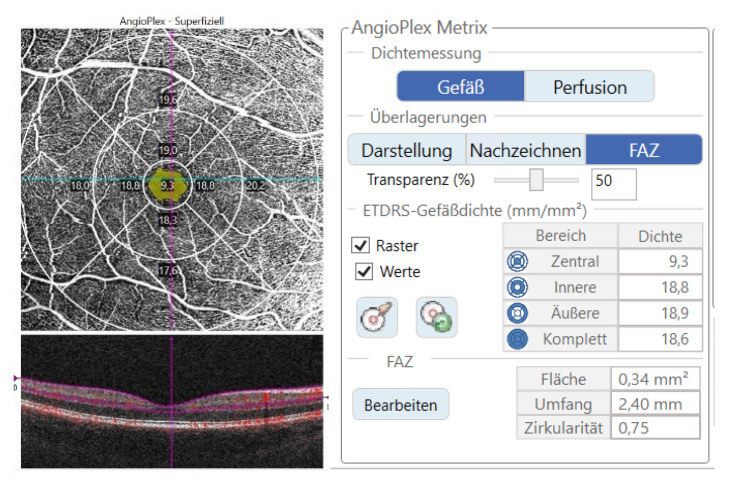
Macular OCT-A 6 × 6 mm^2^ scan analyzed using AngioPlex Metrix. The software interface includes *Gefäß (vessel)* and *Perfusion (perfusion)* display modes with optional *Überlagerungen (overlays)* and adjustable *Transparenz (transparency, %)*. Quantitative *Dichtemessung (density measurement)* comprises *ETDRS-Gefäßdichte (ETDRS vessel density (VD))* and *ETDRS-Perfusionsdichte (ETDRS perfusion density (PD))*. Results are presented by *Bereich (region)* as *Zentral (central)*, *Innen (inner)*, *Äußere (outer)*, and *Komplett (complete)*, with corresponding *Dichte (density)* values. Additional options include *Raster (grid)* and *Werte (values)* display, as well as *Nachzeichnen (tracing)* and *Bearbeiten (edit)* functions. The *FAZ (foveal avascular zone)* analysis provides *Fläche (area)*, *Umfang (perimeter)*, and *Zirkularität (circularity)* measurements.

1. **Peripapillary vascular analysis:** Radial peripapillary capillary (RPC)-level segmentation within a 4.5 × 4.5 mm^2^ field, quantifying perfusion (PF%) and flow index (FI) across the complete outer region and individual quadrants (Figure 1).
2. **Macular vascular quantification:** Superficial capillary plexus segmentation measuring vessel density (VD) and perfusion density (PD%) using both 3 × 3 mm^2^ and 6 × 6 mm^2^ protocols, with comprehensive foveal avascular zone (FAZ) morphometric analysis. All segmentation outputs were reviewed manually for obvious errors prior to inclusion in the analysis (Figure 2 and Figure 3).

### 2.6. Statistical Analysis

#### 2.6.1. Overview

Data were analyzed in R (version 4.5.0; R Foundation for Statistical Computing, Vienna, Austria). Continuous variables are presented as mean ± SD, and categorical variables as frequencies/percentages. Normality was assessed with the Shapiro–Wilk test. Group comparisons (children vs. adults) used Student’s *t*-test or Mann–Whitney U test for continuous data and Fisher’s exact test for categorical data, with *p* < 0.05 indicating nominal significance.

#### 2.6.2. Univariable Correlation Framework

To investigate relationships between all measured parameters, a comprehensive correlation analysis was performed between demographic, clinical, biometric, OCT, and OCT-A parameters. Statistical significance was primarily determined by *p*-values, with correlations considered significant at *p* < 0.001 to control for multiple comparisons. A significance threshold of *p* < 0.001 was chosen a priori as a conservative criterion to reduce false-positive findings, given the exploratory, hypothesis-generating nature of this analysis. Given the large number of statistical tests performed, a conservative significance threshold of *p* < 0.001 was predefined a priori. In a post hoc sensitivity analysis, false discovery rate (FDR) adjustment of all conducted tests showed that an unadjusted *p* = 0.001 corresponded approximately to an FDR-adjusted *p* = 0.0017, supporting the adequacy of the chosen threshold. Nevertheless, the analysis should be interpreted as exploratory, and the findings require independent replication.

To quantify the correlation between various parameters, we computed the pseudo R^2^ using McFadden’s definition R^2^ = 1 − ‘model deviance’/’null deviance’, for the univariable generalized linear model (GLM). Here, the model is selected according to the dependent variable: Gaussian LM (continuous variables) and logistic GLM (dichotomous variables). The correlation coefficient was then derived by rho = sign(beta) × √R^2^, with beta representing the model parameter. For pairs of continuous variables, this GLM-based rho is numerically equivalent to the Pearson correlation coefficient. This parameter comparison includes demographic characteristics (age, gender), clinical findings, biometric measurements (AL, spherical equivalent), and all OCT/OCT-A. This generalized GLM-based framework was initially implemented to allow consistent handling of variables with different outcome types. However, all associations reported and interpreted in the final analyses were derived from Gaussian linear models. Consequently, the reported *R*^2^ values correspond to conventional linear-model *R*^2^ measures, and the derived correlation coefficients are equivalent to standard Pearson-type correlations. To prioritize correlations based on biological plausibility and clinical relevance, we assigned priority scores to potential correlations between parameter groups (Table 1). Each parameter group combination was evaluated and assigned a priority score, where score 5 indicated highly probable and clinically relevant relationships, score 4 represented moderately probable relationships with an established anatomical basis, and score 0 indicated unlikely or non-clinically relevant associations. Only correlations with priority scores of 4 or 5 were included in the prioritized distribution analysis to focus on the most clinically meaningful relationships.

Correlation magnitude was classified as small (|rho| = 0.10–0.29), medium (|rho| = 0.30–0.49), large (|rho| = 0.50–0.79), or very large (|rho| ≥ 0.80) according to Cohen’s guidelines [6]. Statistical significance was determined first using *p*-values, then correlation strength was assessed using rho values to interpret the clinical relevance of significant relationships.

#### 2.6.3. Between-Group Comparison of Correlation Coefficients

To formally assess differences in correlation structure between pediatric and adult participants, group-specific correlation coefficients were directly compared using Fisher’s r-to-z transformation. Pearson-equivalent correlation coefficients derived from the LM-based framework were transformed to z-scores and compared between groups using standard two-tailed tests. Only associations with significant Fisher r-to-z comparisons (*p* < 0.001) were interpreted as reflecting genuine age-group differences in correlation structure.

#### 2.6.4. Multivariable Analysis with Adjustment for Confounders

To validate our initial correlation findings, we examined whether significant associations from the univariable analysis persisted after adjusting for confounders. This approach helps distinguish true biological relationships from artifacts caused by ocular magnification or other biometric factors, particularly important when analyzing correlations across pediatric and adult populations, where eye growth and maturation may confound measurements. Zhang et al. demonstrated that age, AL, body mass index, systolic and diastolic blood pressure, central corneal thickness, and intraocular pressure significantly influenced RNFL thickness measurements in children aged 6–8 years [7]. Their findings revealed that without appropriate adjustment for these variables, particularly AL-related magnification effects, pediatric RNFL thickness measurements could be systematically overestimated. Complementarily, Ahn et al. reported substantial effects of refractive error and AL variations on RNFL measurements across pediatric and adolescent populations, underscoring the necessity for statistical adjustment when conducting cross-age group comparisons [8].

We employed generalized linear models to examine the independent effects of variables on OCT and OCT-A parameters while controlling for known confounders. Based on the available data in our cohort and following the methodology of Zhang et al., the following variables were included in the multivariable models: age (continuous variable), gender (binary: male/female), intraocular pressure (IOP, mmHg), and axial length (AL IOL Master, mm). Covariates were selected a priori based on published evidence of their influence on retinal structural and vascular parameters. No post hoc or stepwise variable selection was performed. For each parameter pair showing a significant univariable association, multivariable linear regression models were fitted to assess associations while accounting for potential confounding effects and age-group-specific relationships according to the following formula:

Parameter 1 ~ Parameter 2 × Age-Group + Age + Gender + IOP + Axial Length

where Parameter 1 represents the dependent variable and Parameter 2 the independent variable of interest, with age (continuous, years), gender (binary), intraocular pressure (continuous, mmHg), and axial length (continuous, mm) as pre-specified covariates. The model family was selected according to the distribution of the dependent variable: Gaussian for continuous outcomes, binomial for dichotomous outcomes, and multinomial for multi-categorical outcomes. Statistical significance of Parameter 2 in the multivariable model was assessed at *p* < 0.001, consistent with the univariable threshold.

The multivariable model was applied to the entire cohort (children and adults combined, n = 65) rather than within age subgroups, owing to sample size limitations and degrees of freedom considerations. Given our sample size of 65 participants, we focused on confirming whether correlations observed in univariable analysis remained significant in the adjusted models, providing additional confidence in our findings while acknowledging the statistical power limitations of complex multivariable models.

For multivariable models, a *p*-value < 0.001 was considered statistically significant to maintain consistency with the univariable analysis threshold. As with the univariable analysis, we calculated the regression-derived correlation coefficient, while statistical significance was determined by *p*-values. Model quality was assessed using R^2^, representing the explained variance of the full multivariable model. For interpretation of effect sizes, R^2^ values of 0.01, 0.09, and 0.25 correspond to small, medium, and large effects, respectively, following Cohen’s conventions [6]. Notably, an R^2^ of 0.25 is equivalent to a correlation coefficient (r) of 0.5 in univariable analysis, representing a large effect size. Higher R^2^ values (approaching 1) indicate better model fit. R^2^c denotes the variance explained by the confounder-only model (including age, gender, IOP, and axial length but excluding the parameter of interest), allowing assessment of the incremental explanatory value of the primary association beyond confounders. Nested models (confounder-only model vs. full model including the parameter of interest and interaction term) were compared using ANOVA-based F-tests to assess the incremental explanatory value of the primary association beyond confounders.

#### 2.6.5. Group-Wise Linkage Proportion

Due to the high number of significant correlations identified in the univariable analysis, we implemented a prioritization strategy to focus on clinically relevant parameter relationships. Clinical, biometric, OCT, and OCT-A parameters were categorized into eight predefined groups based on anatomical location and measurement type: Demographics, Biometry, Clinical findings, Macular vascular, FAZ parameters, Papillary vascular, Papillary thickness, and Macular thickness.

To summarize statistical correlations between parameter groups, we introduce the ‘Group-wise Linkage Proportion’. Therefore, the percentage of potential significant correlations (*p* < 0.05) within each parameter category combination for children versus adults was computed. This approach allowed for direct comparison of correlation patterns between age groups while focusing on biologically plausible and clinically relevant associations. It should be noted that correlations between parameter groups may exhibit directional asymmetry, as the correlation of a categorical factor against an interval-scaled random variable represents a different statistical model than the reverse relationship, potentially resulting in different significance patterns.

This form of analysis provides insight into which anatomical domain relationships show preferential association patterns in pediatric versus adult populations, potentially reflecting developmental or age-related shifts in retinal integration architecture among the most clinically relevant parameter combinations.

## 3. Results

### 3.1. Study Cohort

The cohort comprised 37 pediatric participants (mean 10.20 ± 4.42 years) and 28 adults (mean 37.78 ± 12.49 years) (Table 2). Gender distribution was balanced in the pediatric group (56.8% female) but showed a pronounced female predominance in adults (82.1%; *p* > 0.05). AL differed significantly between groups, with adults demonstrating longer eyes than children (23.84 ± 0.84 mm vs. 22.90 ± 0.99 mm; *p* < 0.001). Spherical equivalent refraction also differed (*p* < 0.05), adults tending towards myopia (−1.125 ± 2.14 diopters) compared with children (−0.146 ± 1.81 diopters).

Best-corrected visual acuity (BCVA) was excellent in both cohorts and did not differ significantly (LogMAR 0.017 ± 0.045 in children vs. 0.004 ± 0.019 in adults; *p* > 0.05). IOP remained within normal limits across the sample with no significant group difference (16.86 ± 3.79 mmHg in children vs. 15.64 ± 3.95 mmHg in adults; *p* = 0.211). Pupil diameter measurements revealed significant differences between pediatric age subgroups, with younger children showing smaller pupils compared to older children: 5.321 ± 1.018 mm versus 6.254 ± 1.103 mm (*p* = 0.020). Adult subgroups showed no significant pupil diameter differences (18–31 years: 5.564 ± 0.868 mm vs. >32 years: 4.764 ± 1.566 mm; *p* = 0.107).

### 3.2. OCT Global Parameters (Table 3)

OCT signal quality showed significant age-related differences within the pediatric group for optic disc measurements, with younger children (1–9 years) demonstrating superior signal quality compared to older children (10–17 years): 9.261 ± 1.054 versus 8.286 ± 0.914 (*p* = 0.004). Macular signal quality remained comparable across all age subgroups. Adult subgroups showed no significant differences in either optic disc or macular signal quality, indicating stable image reliability within the adult cohort. Average RNFL thickness and vertical cup-to-disc ratio likewise did not differ significantly between pediatric and adult cohorts. In contrast, macular cube total thickness was markedly greater in adults than in children (270.36 ± 17.02 μm vs. 251.67 ± 21.32 μm; *p* < 0.001), whereas GCIPL total thickness remained similar across cohorts (83.15 ± 5.43 μm vs. 83.83 ± 5.80 μm; *p* > 0.05).

### 3.3. OCT-A Parameters (Table 4)

Signal quality for OCT-A was adequate in both cohorts, supporting reliable vascular quantification. Optic nerve head (ONH) perfusion was higher in the pediatric group, with ONH PF (complete outer region) 45.74 ± 1.66% in children versus 44.33 ± 1.26% in adults (*p* < 0.001), whereas FI measures did not differ significantly between groups.

Macular VD and PD were broadly comparable across most metrics, and foveal avascular zone measures showed no meaningful age differences: FAZ area 0.228 ± 0.100 mm^2^ (children) vs. 0.212 ± 0.071 mm^2^ (adults), FAZ perimeter 1.875 ± 0.499 mm vs. 1.850 ± 0.323 mm, and FAZ circularity 0.773 ± 0.076 vs. 0.748 ± 0.090 (all *p* > 0.05). Overall, apart from higher ONH perfusion in children, macular microvascular architecture appeared comparable between healthy pediatric and adult eyes.

### 3.4. Univariable Correlation Analysis

A total of 90 univariable correlations met the pre-specified significance threshold (*p* < 0.001), revealing markedly different correlation architectures between pediatric and adult eyes.

#### 3.4.1. Cross-Group Correlations (Both Children and Adults, n = 14)

Across both cohorts, 14 cross-group correlations were highly consistent, dominated by three themes (detailed results in Table 5). FAZ metrics showed very large negative correlations with VD measures across scan protocols and regions, with coefficients spanning approximately r = −0.67 to −0.87 in adults and r = −0.55 to −0.70 in children, a pattern that held for both 3 × 3 and 6 × 6 acquisitions and for central and parafoveal zones.

Macular VD parameters correlated robustly and positively with structural macular thickness, particularly with macular cube total thickness, producing large effect sizes (all r > 0.50).

ONH FI measures related strongly and positively to RNFL thickness across multiple quadrants (nasal, temporal and inferior), and clock-hour positions.

#### 3.4.2. Adult-Specific Correlations (n = 36)

Age-specific networks diverged substantially (detailed results in Table 5). In adults, 36 unique correlations were identified. The dominant pattern involved **macular thickness–vascular coupling** (29 correlations), concentrated parafoveally and observed across 3 × 3 and 6 × 6 VD/PD metrics, with moderate to large correlations (r ≈ 0.57–0.70).

Additional adult links included ONH FI/PD associations with RNFL in defined quadrants and clock hours—for example, ONH FI temporal correlated with RNFL superior (r = 0.69) and RNFL hour 12 (r = 0.66), while ONH PD temporal correlated with RNFL hour 8 (r = 0.64) and the temporal quadrant (r = 0.675).

Macular FI yielded only a single moderate positive correlation (3 × 3 VD parafoveal temporal with GCIPL temporal-superior, r = 0.581), and FAZ measures in 6 × 6 scans produced strong negative (FAZ area vs. 6 × 6 VD central, r = −0.772) and moderate positive (FAZ perimeter vs. 6 × 6 PD perifoveal inferior, r = 0.588) associations.

#### 3.4.3. Pediatric-Specific Correlations (n = 40)

The pediatric network comprised 40 unique correlations and was characterized by ONH perfusion–RNFL connectivity and biometric influences (detailed results in Table 5). Clinical variables produced ten notable links: AL showed multiple medium-to-large negative correlations (r ≈ −0.53 to −0.61) with average RNFL, specific RNFL quadrants, and several ONH FI metrics. Age correlated moderately and negatively with ONH PF inferior (r = −0.565), and spherical equivalent exhibited moderate positive correlations with ONH FI parameters (r ≈ 0.54–0.56).

The largest pediatric cluster comprised 25 ONH FI/PF–RNFL correlations, where ONH FI nasal demonstrated very large positive connectivity (r ≈ 0.54–0.77) across multiple RNFL sectors. The outer region of ONH FI showed moderate to large positive links (r ≈ 0.54–0.63) with quadrants and clock hours, and ONH PF parameters (outer, nasal, superior) correlated moderately to strongly (r ≈ 0.55–0.75) with average and regional RNFL thickness.

FAZ/macular correlations (3 correlations) involved strong negative relationships between FAZ parameters and 3 × 3 PD central measurements (r = −0.674 to r = −0.708), as well as a large positive correlation between 3 × 3 PD central and total MT (r = 0.648).

### 3.5. Multivariable Correlation Analysis

After adjusting for age, gender, IOP, and AL, 52 of the original 90 univariable correlations remained statistically significant (*p* < 0.001), thereby confirming that a substantial subset of the observed associations is independent of these key confounders. The multivariable modelling revealed notable coherencies across different parameter categories, with 14 correlations reaching large effects as defined by R^2^ ≥ 0.25.

### 3.6. Cross-Group Correlations Confirmed

The cross-group analysis demonstrates exceptional stability: **all 14** correlations that were significant in both children and adults in univariable testing persisted following multivariable adjustment, indicating robust age-independent relationships. This complete confirmation rate (100%) underscores the biological robustness of these shared associations, which include FAZ–VD relationships, macular VD–thickness coupling, and ONH FI–RNFL connectivity.

The most powerful adjusted associations cluster around FAZ–VD relationships, with FAZ area versus 3 × 3 VD central producing the highest R^2^ of 0.575 (R^2^c = 0.039, *p* < 0.0001). Complementary strong effects included FAZ perimeter versus 3 × 3 VD central (R^2^ = 0.538, R^2^c = 0.034, *p* < 0.001) and FAZ perimeter versus macular cube total thickness (R^2^ = 0.414, R^2^c = 0.035, *p* < 0.0001).

Macular VD measures preserved strong multivariable links with structural thickness endpoints. The correlation between 3 × 3 VD central and macular cube total thickness exemplifies this stability and produced a very large effect (R^2^ = 0.657, R^2^c = 0.001, *p* < 0.0001).

ONH haemodynamic indices largely retained their univariable associations with RNFL thickness after confounder control. Notably, ONH FI nasal versus average RNFL thickness achieved an R^2^ of 0.677 (R^2^c = 0.315, *p* < 0.0001).

### 3.7. Age-Specific Correlations After Adjustment

Within the pediatric subset, 23 of 40 previously observed age-specific correlations remained significant after multivariable adjustment, indicating that the majority of pediatric univariable associations were robust to confounding.

The multivariable resilience in children clustered around nasal ONH perfusion parameters: ONH FI nasal preserved very large adjusted effects with the RNFL superior quadrant (R^2^ = 0.635, R^2^c = 0.315, *p* < 0.0001), hour 1 (R^2^ = 0.615, R^2^c = 0.315, *p* < 0.0001), and hour 12 (R^2^ = 0.541, R^2^c = 0.315, *p* < 0.0001). Likewise, ONH PF nasal demonstrated robust adjusted associations with the RNFL nasal quadrant (R^2^ = 0.515, R^2^c = 0.302, *p* < 0.001) and with specific clock-hours such as hour 2 (R^2^ = 0.518, R^2^c = 0.302, *p* < 0.001).

Adult networks proved less stable under multivariable scrutiny: 15 of 36 adult-specific correlations (41.7%) remained significant after adjustment. The strongest adult-adjusted effects centred on macular thickness relationships to VD metrics, several of which reached medium to large R^2^ values as detailed in Table 5.

### 3.8. Group-Wise Linkage Proportion (Table 6)

Given the extensive number of significant correlations identified, we performed the exploratory group-wise linkage analysis described in the Section 2 to summarize higher-level association patterns between parameter categories. This analysis indicated differing correlation patterns between pediatric and adult participants.

**Table 6:** Group-wise Linkage Proportions between children and adults. Values represent the proportion of significant correlations (*p* < 0.05) relative to the total number of tested parameter pairs within each group-pair combination, displayed as Children/Adults. Color coding: blue = children, red = adults. This exploratory analysis provides a descriptive summary of correlation patterns across parameter categories.

Parameter Groups	Demographics	Biometry	Clinical Findings	Macular Vascular	FAZ	Papillary Vessels	Papillary Thickness	Macular Thickness
Demographics	-	**14.3%** **/28.6%**	**16.7%** **/16.7%**	**18.4%** **/2.6%**	**0.0%** **/0.0%**	**10.0%** **/0.0%**	**5.6%** **/11.1%**	**3.1%** **/3.1%**
Biometry	**14.3%** **/28.6%**	-	**0.0%** **/0.0%**	**15.0%** **/1.5%**	**0.0%** **/14.3%**	**31.4%** **/15.7%**	**15.9%** **/7.9%**	**13.4%** **/1.8%**
Clinical Findings	**16.7%** **/16.7%**	**0.0%** **/0.0%**	-	**0.0%** **/2.6%**	**0.0%** **/0.0%**	**6.7%** **/0.0%**	**7.4%** **/0.0%**	**10.4%** **/0.0%**
Macular Vascular	**21.1%** **/2.6%**	**15.0%** **/1.5%**	**0.0%** **/2.6%**	-	**8.8%** **/21.9%**	**14.2%** **/31.1%**	**5.3%** **/20.8%**	**1.2%** **/48.4%**
FAZ	**0.0%** **/0.0%**	**0.0%** **/14.3%**	**0.0%** **/0.0%**	**8.8%** **/21.9%**	-	**0.0%** **/10.0%**	**0.0%** **/3.7%**	**4.2%** **/4.2%**
Papillary Vascular	**15.0%** **/0.0%**	**31.4%** **/15.7%**	**6.7%** **/0.0%**	**14.2%** **/31.1%**	**0.0%** **/10.0%**	-	**51.1%** **/29.4%**	**35.6%** **/32.5%**
Papillary Thickness	**5.6%** **/8.3%**	**15.9%** **/7.9%**	**7.4%** **/0.0%**	**5.3%** **/20.8%**	**0.0%** **/3.7%**	**51.1%** **/29.4%**	-	**51.7%** **/37.2%**
Macular Thickness	**3.1%** **/3.1%**	**13.4%** **/1.8%**	**10.4%** **/0.0%**	**1.2%** **/48.4%**	**4.2%** **/4.2%**	**35.6%** **/32.5%**	**51.7%** **/37.2%**	-

Abbreviations: FAZ, foveal avascular zone.

### 3.9. Pediatric Correlation Architecture

Children showed five group pairs with linkage proportions exceeding 20%:

- Papillary thickness ↔ Macular thickness: 51.7%;
- Papillary vascular ↔ Papillary thickness: 51.1%;
- Papillary vascular ↔ Macular thickness: 35.6%;
- Biometry ↔ Papillary vascular: 31.4%;
- Macular vascular ↔ Demographics: 21.1%.

### 3.10. Adult Correlation Architecture

Adults demonstrated greater correlation diversity with eight group pairs exceeding 20% linkage proportion:

- Macular vascular ↔ Macular thickness: 48.4%;
- Papillary thickness ↔ Macular thickness: 37.2%;
- Papillary vascular ↔ Macular thickness: 32.5%;
- Papillary vascular ↔ Macular vascular: 31.1%;
- Papillary vascular ↔ Papillary thickness: 29.4%;
- Demographics ↔ Biometry: 28.6%;
- Macular vascular ↔ FAZ: 21.9%;
- Papillary thickness ↔ Macular vascular: 20.8%.

The adult cohort exhibited more complex inter-domain relationships, with macular vascular parameters showing the strongest correlation weight with macular thickness, while pediatric correlations were dominated by papillary-macular structural relationships.

### 3.11. Age-Related Differences in Linkage Proportions

The most notable differences in group-wise linkage proportions were observed in:

- Macular vascular ↔ Macular thickness: 1.2% (children) vs. 48.4% (adults)—a 40-fold increase
- Demographics ↔ Biometry: 14.3% (children) vs. 28.6% (adults)—doubling in strength
- Macular vascular ↔ FAZ: 8.8% (children) vs. 21.9% (adults)—2.5-fold increase
- Papillary vascular ↔ Macular vascular: 14.2% (children) vs. 31.1% (adults)—more than doubling.

These exploratory findings suggest potential age-related differences in correlation architecture, although formal statistical comparison of linkage proportions was not performed.

## 4. Discussion

### 4.1. Age-Related Differences in Retinal Correlation Patterns

Our analysis reveals clear age-associated differences in retinal parameter correlation patterns, with potential implications for clinical interpretation and the development of normative reference standards. Both pediatric and adult cohorts retain core structural linkages, yet adults acquire distinct vascular-centred patterns that are largely absent in children.

One of the most notable findings was the substantially higher group-wise linkage proportion between macular vascular and macular thickness parameters in adults (48.4%) compared with children (1.2%). This exploratory finding suggests age-related differences in structural–vascular association patterns within the retina, although the cross-sectional design does not allow mechanistic conclusions regarding retinal autoregulatory processes.

### 4.2. Regional Specificity and External Validation

The adult macular vascular–thickness correlations were concentrated in the parafoveal region, particularly in the superior and nasal sectors, mirroring known anatomical distributions where thickness and ganglion cell density are greatest [9,10]. This spatial concordance lends mechanistic plausibility to the findings: regions with higher ganglion cell density demand greater perfusion, and a mature autoregulatory network enables vascular metrics (VD, PD) to reflect tissue volume reliably. Our adult findings align with prior OCT-A studies: Yu et al. reported robust parafoveal VD–inner retinal thickness correlations in adults (mean age 38 ± 13 years) with significance emerging predominantly in subjects older than 35, supporting the view of post-adolescent vascular–structural maturation [11]. Park et al. reported comparable macular VD–GCIPL associations [12]. These convergent findings from independent cohorts and technologies strengthen the external validity of our adult linkage architecture.

### 4.3. Pediatric Contrasts and Developmental Timing

Contrastingly, pediatric studies generally report weak, inconsistent, or even inverse macular thickness–VD relationships. Comba et al. found no significant foveal thickness–VD correlations in superficial or deep plexuses [13]; Içel et al. observed an inverse relationship whereby superficial and deep VD decreased as central macular thickness increased [14]; Karakucuk et al. detected associations restricted to the deep plexus [15]; and Bajtl et al. reported no significant thickness–vascular correlations across layers [16]. These heterogeneous pediatric results, together with our finding of dominant papillary–macular structural weights in children (papillary thickness ↔ macular thickness 51.7%), suggest that structural lamination is established early, whereas vascular–structural integration follows a protracted postnatal course. Developmental biology provides a plausible framework for interpreting these findings: foveal lamination attains early morphological completion [17], whereas vascular maturation [18] and autoregulatory capacity [19] are reported to develop over years after birth.

Thus, structural templates appear to precede vascular consolidation: the retina first secures anatomic architecture, and only later does perfusion become tightly coupled to that structure. Longitudinal evidence supports this view: Yamashita et al. reported that correlations between axial length and RNFL were absent at 8–9 years but became significant by 14–15 years, indicating that key parameter relationships emerge progressively through adolescence rather than being present from birth [20].

### 4.4. Structural Correlations and FAZ Stability

Both age groups exhibited strong papillary–macular thickness correlations (children: 51.7%, adults: 37.2%), consistent with retinal anatomy, in which ganglion cell axons from the macula converge at the optic nerve head. This dominant structural link in children is consistent with the hypothesis that anatomical coherence is established early, a pattern that would mirror embryonic retinal development, in which structural templates form before vascular networks mature. However, our cross-sectional design cannot establish this developmental sequence directly [21]. The relatively higher structural weight in the pediatric cohort supports the notion that tissue morphology is established early, while vascular-structural integration evolves postnatally [22], in line with the “plate hypothesis” and longitudinal observations of progressive remodelling between 8 and 15 years [20].

The consistent presence of FAZ-VD correlations in both age groups (r = −0.55 to −0.87) confirms a fundamental anatomical relationship that is preserved throughout development. In our multivariable models, these associations remained independent of age, gender, IOP and axial length, exhibiting the largest effects observed (R^2^ up to 0.58). This finding accords with the early maturation of foveal structure: the FAZ is an architecturally fixed feature of the fovea that is established early in development and thus provides a reliable reference for macular perfusion measurements [22].

### 4.5. Multivariable Analysis

Multivariable adjustment supported the biological relevance of the majority of identified associations: 58% of significant correlations (52 of 90) remained statistically significant after adjusting for age, gender, IOP, and axial length. Pediatric correlations showed greater stability under multivariable adjustment than adult correlations: with 57.5% of pediatric-specific correlations (23 of 40) remaining significant compared with 41.7% of adult-specific correlations (15 of 36).

This differential stability pattern has potential biological implications. The greater robustness of pediatric correlations to confounder adjustment suggests that the ONH perfusion–RNFL relationships observed in children may represent more direct anatomical coupling.

In contrast, the adult-specific macular vascular–thickness correlations appear more susceptible to confounding by biometric and demographic factors. One possible interpretation is that pediatric correlation patterns reflect fundamental structural relationships established during retinal development, while adult-specific correlations—particularly the macular vascular–thickness coupling—emerge within a more complex, interdependent system where multiple factors jointly influence the observed associations.

The finding that adult correlations are more vulnerable to confounder adjustment also aligns with the nature of the correlations themselves. Adult-specific associations were dominated by macular vascular–thickness coupling concentrated in specific parafoveal sectors (superior and nasal), which may be more sensitive to variations in AL and associated magnification effects than the broader ONH–RNFL relationships that characterize pediatric patterns.

AL showed univariable associations with multiple OCT/OCT-A parameters in children that frequently failed multivariable confirmation, consistent with dynamic structural remodelling during growth [20]. Likewise, many ONH flow-index correlations with RNFL clock-hour measures lost significance after adjustment (17 of 40 in children, 21 of 36 in adults), implying that those unadjusted associations partly reflect developmental or biometric confounders rather than exclusively anatomical coupling. By contrast, FAZ correlations persisted after adjustment in both age groups, reinforcing their early and independent establishment [10]. The heterogeneity of effect sizes across parameter pairs is itself a meaningful finding. Core anatomical relationships—such as FAZ geometry with central VD, or ONH flow index with RNFL thickness—consistently produce large-to-very-large effects in both age groups (R^2^ > 0.40, equivalent to r > 0.63), reflecting direct structural-vascular coupling with established anatomical substrate. By contrast, associations involving peripheral macular sectors or less anatomically proximate parameter pairs naturally yield smaller effects, consistent with greater biological variability and more distal structural relationships. This graded effect-size structure supports the biological plausibility of the findings rather than undermining it.

Notably, the cross-group correlations (those significant in both children and adults) demonstrated exceptional stability: 14 of 14 (100%) remained significant after multivariable adjustment. This striking robustness suggests that these shared correlations—primarily FAZ–VD relationships and ONH FI–RNFL associations—represent fundamental, age-independent anatomical relationships that are minimally influenced by the confounders examined. In contrast, age-specific correlations were more susceptible to adjustment, with adult-specific correlations showing the greatest vulnerability (only 41.7% confirmed vs. 57.5% in children). This pattern suggests that while core retinal structure–function relationships are established early and remain stable across the lifespan, the age-specific correlation patterns identified in our analysis may partly reflect developmental stage-dependent interactions between biometric factors and retinal parameters. The subset of 52 associations confirmed by multivariable analysis represents the clinically informative core of our findings and most directly supports our conclusions regarding age-specific retinal integration patterns.

### 4.6. Between-Group Differences in Correlation Structure

Fisher r-to-z transformation identified statistically significant differences in correlation strength between age groups for several parameter pairs (Table 5). Correlations between FAZ and VD-related parameters tended to be stronger in adults, while ONH FI–RNFL correlations were more pronounced in children (e.g., ONH FI nasal vs. average RNFL: r = 0.872 in children vs. r = 0.620 in adults; Fisher z *p* < 0.05). These formal comparisons are consistent with differing correlation patterns between pediatric and adult retinas, although the exploratory nature of the analysis warrants cautious interpretation.

### 4.7. Limitations

Several limitations should be considered when interpreting these findings. The cross-sectional design provides snapshots rather than developmental trajectories; therefore, causal timing must be inferred from comparative and external longitudinal data rather than observed directly. Unequal group sizes may influence statistical power for detecting age-specific effects, although our prioritised linkage approach mitigates some imbalance. A further limitation is the high-dimensional nature of the dataset relative to the cohort size. With 37 pediatric and 28 adult participants and a large number of OCT/OCT-A parameters assessed, correlation estimates may be statistically unstable and disproportionately influenced by individual observations or minor variations in sample composition. This instability is particularly relevant for subgroup-specific correlations, where effective sample sizes are smaller. Findings should therefore be interpreted with appropriate caution, and the present results are best regarded as exploratory effect-size estimates requiring replication.

An interesting methodological finding was the paradoxical relationship between pupil diameter and OCT signal quality in children: younger children with smaller pupils (5.3 mm) demonstrated better optic disc signal quality than older children with larger pupils (6.3 mm). This unexpected finding suggests that factors beyond simple optical considerations influence pediatric OCT image quality, possibly including cooperation, fixation stability, or accommodation.

Although this did not affect our primary correlation analyses, it highlights the complexity of pediatric retinal imaging optimization. Residual confounding remains possible despite extensive adjustment. The study population consisted exclusively of healthy Caucasian volunteers recruited from a single centre. OCT and OCT-A parameters are known to vary across ethnic groups, and the reported correlation structures may not be directly generalisable to non-Caucasian populations or to multicentre settings with different demographic compositions and acquisition protocols. Additionally, the adult group showed a higher proportion of female participants compared to the pediatric group. Although sex was included as a covariate in all multivariable analyses, this sex imbalance between age groups represents a potential source of residual confounding that should be considered when interpreting the reported between-group differences in correlation structure.

All data were acquired using a single Zeiss CIRRUS HD-OCT 5000 AngioPlex device at a single centre, which ensures complete internal consistency but limits cross-platform generalisability. The reported correlation structures may differ when assessed on alternative platforms that employ different segmentation algorithms, axial resolutions, or OCT-A flow detection methods. Replication across diverse imaging platforms and multicentre settings is therefore necessary before these findings can be translated into broadly applicable clinical reference frameworks.

Finally, our analysis targeted a predetermined set of anatomically plausible correlation pairs. Unbiased matrix-based or machine-learning approaches, such as random forest or support vector machines, may uncover additional, possibly subtler, age-specific relationships in larger datasets and represent a valuable direction for future investigation.

The distinction between statistical and practical significance warrants explicit acknowledgement. Of the 90 univariable correlations meeting *p* < 0.001, a subset showed correlation coefficients in the medium range (|rho| = 0.50–0.65), which, although statistically robust at this threshold, may not individually carry sufficient effect size for direct clinical decision-making. The present study was designed as an exploratory hypothesis-generating correlation analysis rather than a predictive or diagnostic framework. Accordingly, the reported associations should be interpreted as identifying candidate relationships that merit prospective validation in larger, independent cohorts before clinical reference standards can be derived.

## 5. Conclusions

This study provides a comprehensive comparison of retinal structural–vascular integration between healthy children and adults using standardized OCT and OCT-A protocols. Our findings demonstrate that, although core anatomical relationships—particularly FAZ geometry with central VD and ONH perfusion with peripapillary RNFLare conserved across age groups, the broader correlation architecture differs substantially.

Children exhibit a correlation network dominated by ONH perfusion–RNFL coupling, whereas adults show a shift in emphasis toward macular vascular–thickness integration that is largely absent in pediatric eyes.

Multivariable adjustment revealed that pediatric correlations demonstrate greater robustness to confounding than adult-specific associations: 57.5% of pediatric correlations remained significant after adjustment compared with only 41.7% of adult correlations. Notably, correlations present in both age groups showed complete stability (100%), indicating that these shared associations represent fundamental, age-independent structural–vascular relationships.

These age-specific differences have clinical implications for both diagnostic interpretation and disease monitoring. In pediatric populations, where the ONH–RNFL network predominates, disruptions in peripapillary perfusion–structure relationships may serve as early biomarkers for conditions affecting the optic nerve. In adults, the emergence of macular integration patterns suggests that foveal and parafoveal metrics may provide complementary diagnostic information for conditions such as age-related maculopathies and diabetic retinopathy.

In summary, pediatric and adult retinas exhibit fundamentally different structural–vascular correlation architectures. These findings should inform the development of age-appropriate reference standards and may enhance early detection of retinal and optic nerve pathology across the lifespan.

## Figures and Tables

**Table 1:** Priority matrix for correlation analysis. Matrix values represent assigned priority scores for correlations between parameter groups. Higher priority scores indicate stronger expected biological correlations between parameter groups. Note: Correlation of a categorical factor against an interval-scaled random variable represents a different statistical model than the reverse relationship.

Parameter Groups	Demographics	Biometry	Clinical Findings	Macular Vascular	FAZ	Papillary Vascular	Papillary Thickness	Macular Thickness
Demographics	0	0	0	5	5	5	5	5
Biometry	0	0	0	5	5	5	5	5
Clinical Findings	0	0	0	5	5	5	5	5
Macular Vascular	5	5	5	0	4	0	0	4
FAZ	5	5	5	4	0	0	0	4
Papillary Vascular	5	5	5	0	0	0	4	0
Papillary Thickness	5	5	5	0	0	4	0	0
Macular Thickness	5	5	5	4	4	0	0	0

Abbreviations: FAZ, foveal avascular zone.

**Table 2:** Study population demographics and ocular characteristics. Values presented as mean ± SD or n (%). Age subgroups: children (1–9 vs. 10–17 years), adults (18–31 vs. ≥32 years). *p*-Values represent intragroup (children/adults) and intergroup (children vs. adults) comparisons. Statistically significant *p*-values (*p* < 0.05) are highlighted in yellow.

Description	Unit	n.child	Children (Overall)	1–9 Years	10–17 Years	*p*-Value (Children)	n.adult	Adults (Overall)	18–31 Years	≥32 Years	*p*-Value (Adults)	*p*-Value (Overall)
**Optic Nerve Head Analysis**												
Age	Years	37	10.20 ± 4.42 (n = 37)	7.25 ± 1.72 (n = 23)	15.05 ± 2.90 (n = 14)	<0.0001	28	37.78 ± 12.49 (n = 28)	28.87 ± 2.55 (n = 14)	46.70 ± 12.11 (n = 14)	<0.0001	<0.0001
Gender	Female = 1 Male = 2	37	1: 56.8% (n = 21) 2: 43.2% (n = 16)	1: 47.8 (n = 11) 2: 52.3% (n = 12)	1: 71.4% (n = 10) 2: 28.6% (n = 4)	0.288	28	1: 82.1% (n = 23) 2: 17.9% (n = 5)	1: 71.4% (n = 10) 2: 28.6% (n = 4)	1: 92.9% (n = 13) 2: 7.1% (n = 1)	0.326	0.058
Eye	OD = 1 OS = 2	37	1: 36 (97.3%) 2: 1 (2.7%)	1: 22 (95.7%) 2: 1 (4.3%)	1: 14 (100%) 2: 0 (0.0%)	1.000	28	1: 25 (89.3%) 2: 3 (10.7%)	1: 14 (100%) 2: 0 (0.0%)	1: 11 (78.6%) 2: 3 (21.4%)	0.222	0.307
Axial Length	mm	36	22.90 ± 0.99 (n = 36)	22.49 ± 0.757 (n = 22)	23.55 ± 0.981 (n = 14)	<0.001	28	23.84 ± 0.84 (n = 28)	23.826 ± 0.698 (n = 14)	23.853 ± 0.985 (n = 14)	0.934	<0.001
Spherical Equivalent Refraction	dpt	36	−0.146 ± 1.813 (n = 36)	0.068 ± 0.320 (n = 22)	−0.482 ± 1.668 (n = 14)	0.166	28	−1.125 ± 2.144 (n = 28)	−0.2500 ± 0.6124 (n = 14)	−2.000 ± 2.742 (n = 14)	0.132	<0.05
Visual Acuity logMAR	LogMAR	35	0.017 ± 0.045 (n = 35)	0.027 ± 0.046 (n = 22)	0.0000 ± 0.0408 (n = 13)	0.097	28	0.00357 ± 0.01890 (n = 28)	0.00714 ± 0.02673 (n = 14)	0 ± 0 (n = 14)	0.353	0.130
Intraocular Pressure	mmHg	37	16.86 ± 3.79 (n = 37)	17.35 ± 3.59 (n = 23)	16.07 ± 4.12 (n = 14)	0.328	28	15.64 ± 3.95 (n = 28)	16.43 ± 4.15 (n = 14)	14.86 ± 3.72 (n = 14)	0.301	0.211
Pupil size	mm	32	5.700 ± 1.136 (n = 32)	5.321 ± 1.018 (n = 19)	6.254 ± 1.103 (n = 13)	<0.05	28	5.164 ± 1.307 (n = 28)	5.564 ± 0.868 (n = 14)	4.764 ± 1.566 (n = 14)	0.107	0.095

Abbreviations: dpt, diopters; LogMAR, logarithm of minimum angle of resolution; OD, oculus dexter (right eye); OS, oculus sinister (left eye).

**Table 3:** OCT Global Parameters. Values presented as mean ± SD. Age subgroups: children (1–9 vs. 10–17 years), adults (18–31 vs. ≥32 years). *p*-Values represent intragroup (children/adults) and intergroup (children vs. adults) comparisons. Statistically significant *p*-values (*p* < 0.05) are highlighted in yellow.

Description	Unit	n.child	Children (Overall)	1–9 Years	10–17 Years	*p*-Value (Children)	n.adult	Adults (Overall)	18–31 Years	≥32 Years	*p*-Value (Adults)	*p*-Value (Overall)
**Optic Nerve Head Analysis**												
Optic Disc Cube Signal Strength		37	8.892 ± 1.100 (n = 37)	9.261 ± 1.054 (n = 23)	8.286 ± 0.914 (n = 14)	<0.01	28	8.679 ± 0.772 (n = 28)	8.857 ± 0.535 (n = 14)	8.509 ± 0.941 (n = 14)	0.244	0.226
Average RNFL Thickness	μm	37	96.11 ± 11.35 (n = 37)	97.39 ± 9.94 (n = 23)	94.00 ± 13.49 (n = 14)	0.386	28	95.57 ± 8.69 (n = 28)	95.14 ± 8.62 (n = 14)	96.00 ± 9.06 (n = 14)	0.800	0.836
Vertical C/D Ratio		37	0.4116 ± 0.1694 (n = 37)	0.430 ± 0.191 (n = 23)	0.381 ± 0.128 (n = 14)	0.405	28	0.367 ± 0.191 (n = 28)	0.434 ± 0.194 (n = 14)	0.2993 ± 0.1688 (n = 14)	0.060	0.321
Macular Cube Signal Strength		36	9.333 ± 0.894 (n = 36)	9.500 ± 0.802 (n = 22)	9.071 ± 0.997 (n = 14)	0.164	28	9.643 ± 0.678 (n = 28)	9.571 ± 0.756 (n = 14)	9.714 ± 0.611 (n = 14)	0.649	0.121
Macular Cube Total Thickness	μm	36	251.67 ± 21.32 (n = 36)	246.27 ± 16.91 (n = 22)	260.14 ± 25.21 (n = 14)	0.056	28	270.36 ± 17.02 (n = 28)	266.29 ± 14.75 (n = 14)	274.43 ± 18.67 (n = 14)	0.212	<0.001
GCIPL Total	μm	36	83.83 ± 5.80 (n = 36)	83.50 ± 4.62 (n = 22)	84.36 ± 7.47 (n = 14)	0.672	27	83.15 ± 5.43 (n = 27)	84.43 ± 6.09 (n = 14)	81.77 ± 4.44 (n = 13)	0.209	0.635

Abbreviations: C/D, cup-to-disc; GCIPL, ganglion cell–inner plexiform layer; RNFL, retinal nerve fiber layer.

**Table 4:** OCT-A Parameters—Optic Nerve Head and Macular Analysis. Values presented as mean ± SD. Age subgroups: children (1–9 vs. 10–17 years), adults (18–31 vs. ≥32 years). *p*-Values represent intragroup (children/adults) and intergroup (children vs. adults) comparisons. Statistically significant *p*-values (*p* < 0.05) are highlighted in yellow.

Description	Unit	n.child	Children (Overall)	1–9 Years	10–17 Years	*p*-Value (Children)	n.adult	Adults (Overall)	18–31 Years	≥32 years	*p*-Value (Adults)	*p*-Value (Overall)
**Optic Nerve Head Analysis**												
ONH Signal Strength	-	37	9.87 ± 0.41 (n = 37)	9.86 ± 0.36 (n = 14)	0.6628	27	27	9.59 ± 0.74 (n = 27)	9.71 ± 0.61 (n = 14)	9.46 ± 0.88 (n = 13)	0.5070	0.09968
ONH PF - Complete Outer Region	%	37	45.74 ± 1.66 (n = 37)	44.83 ± 1.82 (n = 14)	0.01510	27	27	44.33 ± 1.26 (n = 27)	44.51 ± 1.23 (n = 14)	44.13 ± 1.32 (n = 13)	0.4523	0.0001277
ONH FI - Complete Outer Region	-	37	0.467 ± 0.026 (n = 37)	0.467 ± 0.027 (n = 14)	0.9470	27	27	0.622 ± 0.772 (n = 27)	0.474 ± 0.018 (n = 14)	0.782 ± 1.112 (n = 13)	0.9031	0.2560
**Macular Analysis**												
3 × 3 Signal Strength	Number	35	9.743 ± 0.611 (n = 35)	9.714 ± 0.644 (n = 21)	9.786 ± 0.579 (n = 14)	0.7386	27	9.741 ± 0.594 (n = 27)	9.923 ± 0.277 (n = 13)	9.571 ± 0.756 (n = 14)	0.1620	0.9235
3 × 3 VD Parafoveal Complete	-	35	22.411 ± 1.234 (n = 35)	22.91 ± 0.76 (n = 21)	21.671 ± 1.456 (n = 14)	0.008122	28	22.257 ± 1.640 (n = 28)	22.086 ± 1.865 (n = 14)	22.429 ± 1.430 (n = 14)	0.5898	0.9503
3 × 3 PD Parafoveal Complete	%	35	40.26 ± 2.18 (n = 35)	41.01 ± 1.45 (n = 21)	39.14 ± 2.65 (n = 14)	0.008564	28	39.56 ± 2.72 (n = 28)	39.24 ± 3.06 (n = 14)	39.88 ± 2.41 (n = 14)	0.7474	0.3946
6 × 6 Signal Strength	Number	36	9.69 ± 0.63 (n = 36)	9.82 ± 0.50 (n = 22)	9.51 ± 0.77 (n = 14)	0.2946	28	9.75 ± 0.52 (n = 28)	9.57 ± 0.67 (n = 14)	9.93 ± 0.27 (n = 14)	0.07158	0.9781
6 × 6 VD Parafoveal Complete	-	36	18.40 ± 1.17 (n = 36)	18.62 ± 1.15 (n = 22)	18.06 ± 1.15 (n = 14)	0.04552	28	18.45 ± 0.94 (n = 28)	18.28 ± 1.13 (n = 14)	18.62 ± 0.70 (n = 14)	0.3438	0.8124
6 × 6 PD Parafoveal Complete	%	36	44.25 ± 3.09 (n = 36)	44.75 ± 2.90 (n = 22)	43.46 ± 3.34 (n = 14)	0.1077	28	44.00 ± 2.45 (n = 28)	43.48 ± 2.90 (n = 14)	44.53 ± 1.86 (n = 14)	0.5344	0.3296
6 × 6 PD Perifoveal Complete	%	36	46.18 ± 3.06 (n = 36)	47.05 ± 1.89 (n = 22)	44.81 ± 4.01 (n = 14)	0.03752	28	45.93 ± 2.45 (n = 28)	45.63 ± 2.59 (n = 14)	46.236 ± 2.353 (n = 14)	0.5500	0.5157
6 × 6 VD Perifoveal Complete	-	36	18.50 ± 1.07 (n = 36)	18.83 ± 0.69 (n = 22)	17.99 ± 1.37 (n = 14)	0.03881	28	18.65 ± 0.96 (n = 28)	18.53 ± 1.09 (n = 14)	18.77 ± 0.83 (n = 14)	0.7475	0.5024
**Foveal Avascular Zone (FAZ) Parameters**												
FAZ Area	mm^2^	36	0.228 ± 0.100 (n = 36)	0.219 ± 0.090 (n = 22)	0.242 ± 0.118 (n = 14)	0.5358	27	0.212 ± 0.071 (n = 27)	0.214 ± 0.052 (n = 13)	0.211 ± 0.087 (n = 14)	0.9112	0.4696
FAZ Perimeter	mm	36	1.875 ± 0.499 (n = 36)	1.850 ± 0.478 (n = 22)	1.914 ± 0.546 (n = 14)	0.7119	27	1.850 ± 0.323 (n = 27)	1.889 ± 0.284 (n = 13)	1.814 ± 0.363 (n = 14)	0.5581	0.8186
FAZ Circularity	-	36	0.773 ± 0.076 (n = 36)	0.774 ± 0.070 (n = 22)	0.771 ± 0.087 (n = 14)	0.9093	27	0.748 ± 0.090 (n = 27)	0.753 ± 0.073 (n = 13)	0.7423 ± 0.106 (n = 14)	0.9219	0.1789

Abbreviations: FAZ, foveal avascular zone; FI, flow index; ONH, optic nerve head; PD, perfusion density; PF, perfusion; VD, vessel density.

**Table 5:** This table presents univariable correlations with *p* < 0.001 comparing children versus adults, followed by multivariable analysis results from the combined total group. Only correlations reaching statistical significance at *p* < 0.001 in at least one age group are displayed; Columns show Pearson-equivalent correlation coefficients (rho) and corresponding *p*-values for pediatric and adult participants, respectively. The “Z” column reports the *p*-value of the Fisher r-to-z test for between-group differences in correlation strength. “R^2^” indicates the explained variance of the full multivariable model, whereas “R^2^ Confounder (R^2^c)” denotes the variance explained by the confounder-only model (including age, gender, IOP, and axial length but excluding the parameter of interest). *p* (Model Test) denotes the ANOVA-based F-test comparison between nested models (confounder-only vs. full model). The final column (“Confirmed by multivariable model”) indicates whether the association remained statistically significant (*p* < 0.001) after adjustment for age, gender, intraocular pressure, and axial length.

Parameter 1	Parameter 2	Children		Adults		Z	Total Group—Multivariable Model			
		rho	*p*	rho	*p*		R^2^	R^2^ Confounder (R^2^c)	*p*-Value (ANOVA Model-Test)	Confirmed by Multivariable Model
**Both Groups** ***p*** **< 0.001 (14 Correlations)**										
**FAZ-Parameter Correlations (7 Correlations)**										
FAZ Area	3 × 3 VD central	−0.704	<0.0001	−0.848	<0.0001	0.155	0.575	0.039	<0.0001	Yes
FAZ Area	6 × 6 PD central	−0.631	<0.0001	−0.758	<0.0001	0.342	0.484	0.039	<0.0001	Yes
FAZ Area	Macula Cube Total Thickness	−0.551	<0.001	−0.689	<0.0001	0.390	0.452	0.039	<0.0001	Yes
FAZ Perimeter	3 × 3 VD central	−0.662	<0.0001	−0.870	<0.0001	<0.05	0.538	0.034	<0.0001	Yes
FAZ Perimeter	6 × 6 PD central	−0.658	<0.0001	−0.778	<0.0001	0.341	0.496	0.035	<0.0001	Yes
FAZ Perimeter	6 × 6 VD central	−0.552	<0.001	−0.791	<0.0001	0.084	0.409	0.035	<0.0001	Yes
FAZ Perimeter	Macula Cube Total Thickness	−0.541	<0.001	−0.671	<0.0001	0.430	0.414	0.035	<0.0001	Yes
**Macular Vessel Density/Perfusion Correlations (3 Correlations)**										
3 × 3 VD central	Macula Cube Total Thickness	0.706	<0.0001	0.721	<0.0001	0.910	0.657	0.001	<0.0001	Yes
6 × 6 VD central	Macula Cube Total Thickness	0.547	<0.001	0.655	<0.0001	0.515	0.454	0.037	<0.0001	Yes
6 × 6 PD central	Macula Cube Total Thickness	0.601	<0.0001	0.644	<0.001	0.784	0.509	0.038	<0.0001	Yes
**ONH Flow Index/RNFL Correlations (4 Correlations)**										
ONH FI—Nasal	Average RNFL Thickness	0.872	<0.0001	0.620	<0.001	<0.05	0.677	0.315	<0.0001	Yes
ONH FI—Outer Region	Average RNFL Thickness	0.738	<0.0001	0.671	<0.0001	0.615	0.548	0.274	<0.0001	Yes
ONH FI—Temporal	Average RNFL Thickness	0.562	<0.001	0.652	<0.001	0.586	0.408	0.206	<0.001	Yes
ONH FI—Inferior	Average RNFL Thickness	0.656	<0.0001	0.585	<0.001	0.665	0.488	0.245	<0.0001	Yes
**Adults Only *p* < 0.001 (36 Correlations)**										
**ONH Flow Index & Perfusion Density Correlations (4 Correlations)**										
ONH FI—temporal	RNFL Superior Quadrant	0.430	<0.01	0.690	<0.0001	0.139	0.394	0.206	<0.01	No
ONH FI—temporal	RNFL Hour 12	0.332	<0.05	0.660	<0.0001	0.088	0.367	0.206	<0.01	No
ONH PD—temporal	RNFL Hour 8	0.1812	0.290	0.640	<0.001	< 0.05	0.367	0.147	<0.001	Yes
ONH PD—temporal	RNFL Temporal Quadrant	0.2199	0.197	0.675	<0.0001	< 0.05	0.416	0.147	<0.0001	Yes
**Macula Flow Index Correlations (1 Correlation)**										
3 × 3 VD Parafoveal temporal	GCIPL Temporal Superior	0.059	0.735	0.581	<0.001	<0.05	0.342	0.164	< 0.01	No
**OCT Macula Thickness Correlations (29 Correlations)**										
MT Parafoveal superior	3 × 3 PD Complete	−0.086	0.625	0.610	<0.001	<0.01	0.407	0.227	<0.01	No
MT Parafoveal nasal	3 × 3 PD Parafoveal Complete	−0.040	0.818	0.600	<0.001	<0.01	0.420	0.257	<0.01	No
MT Parafoveal superior	3 × 3 PD Parafoveal Complete	−0.051	0.772	0.639	<0.001	<0.01	0.433	0.227	<0.001	Yes
MT Parafoveal nasal	3 × 3 PD nasal	0.108	0.536	0.597	<0.001	<0.05	0.439	0.257	<0.01	No
MT Parafoveal superior	3 × 3 PD nasal	0.094	0.590	0.641	<0.001	<0.05	0.446	0.227	<0.001	Yes
MT Parafoveal nasal	3 × 3 PD superior	−0.142	0.416	0.690	<0.0001	<0.001	0.439	0.257	<0.01	No
MT Parafoveal superior	3 × 3 PD superior	−0.100	0.567	0.704	<0.0001	<0.001	0.458	0.227	<0.001	Yes
MT Perifoveal superior	3 × 3 PD superior	0.105	0.549	0.600	<0.001	<0.05	0.315	0.160	<0.01	No
MT Parafoveal superior	6 × 6 PD nasal	−0.211	0.216	0.577	<0.001	<0.001	0.410	0.223	<0.01	No
MT Parafoveal superior	3 × 3 VD Complete	−0.021	0.903	0.594	<0.001	<0.01	0.402	0.227	<0.01	No
MT Parafoveal nasal	3 × 3 VD Innere	0.011	0.949	0.583	<0.001	<0.05	0.404	0.257	<0.01	No
MT Parafoveal superior	3 × 3 VD Parafoveal Complete	0.006	0.972	0.647	<0.001	<0.01	0.432	0.227	<0.001	Yes
MT Parafoveal nasal	3 × 3 VD nasal	0.130	0.456	0.590	<0.001	<0.05	0.423	0.257	<0.01	No
MT Parafoveal superior	3 × 3 VD nasal	0.069	0.695	0.644	<0.001	<0.01	0.434	0.227	<0.001	Yes
MT Parafoveal nasal	3 × 3 VD superior	−0.073	0.678	0.590	<0.001	<0.01	0.400	0.257	<0.01	No
MT Parafoveal superior	3 × 3 VD superior	−0.024	0.892	0.637	<0.001	<0.01	0.425	0.227	<0.001	Yes
MT Perifoveal superior	3 × 3 VD superior	0.249	0.149	0.635	<0.001	0.058	0.361	0.160	<0.01	No
MT Parafoveal superior	3 × 3 VD temporal	0.031	0.858	0.592	<0.001	<0.05	0.387	0.227	<0.01	No
MT Parafoveal superior	6 × 6 VD Perifoveal Complete	−0.112	0.516	0.643	<0.001	<0.001	0.444	0.223	<0.001	Yes
MT Parafoveal nasal	6 × 6 VD superior	−0.010	0.952	0.578	<0.001	<0.01	0.391	0.250	<0.01	No
MT Parafoveal superior	6 × 6 VD superior	−0.032	0.855	0.653	<0.0001	<0.01	0.432	0.223	<0.001	Yes
MT Parafoveal nasal	6 × 6 VD Parafoveal Complete	−0.134	0.435	0.600	<0.001	<0.01	0.398	0.250	<0.01	No
MT Parafoveal superior	6 × 6 VD Parafoveal Complete	−0.220	0.197	0.679	<0.0001	<0.0001	0.457	0.223	<0.001	Yes
MT Parafoveal nasal	6 × 6 VD Parafoveal nasal	−0.112	0.517	0.637	<0.001	<0.001	0.420	0.250	<0.01	No
MT Parafoveal superior	6 × 6 VD Parafoveal nasal	−0.222	0.194	0.672	<0.0001	<0.0001	0.451	0.223	<0.001	Yes
MT Parafoveal superior	6 × 6 VD Parafoveal superior	−0.213	0.213	0.573	<0.001	<0.001	0.400	0.223	<0.01	No
MT Parafoveal nasal	6 × 6 VD Parafoveal temporal	−0.022	0.900	0.579	<0.001	<0.01	0.388	0.250	<0.01	No
MT Parafoveal superior	6 × 6 VD Parafoveal temporal	−0.112	0.515	0.653	<0.0001	<0.001	0.439	0.223	<0.001	Yes
MT Parafoveal superior	6 × 6 VD Complete	−0.159	0.363	0.658	<0.0001	<0.001	0.453	0.227	<0.001	Yes
**FAZ-Parameter Correlations (2 Correlation)**										
FAZ Area	6 × 6 VD central	−0.518	<0.01	−0.772	<0.0001	0.084	0.397	0.039	<0.0001	Yes
FAZ Perimeter	6 × 6 PD Perifoveal inferior	0.1480	0.389	0.588	<0.001	<0.05	0.223	0.068	<0.05	No
**Children Only *p* < 0.001 (40 Correlations)**										
**Clinical Parameters (10 Correlations)**										
Age	ONH PF—Inferior	−0.565	<0.001	−0.284	0.135	0.194	0.759	0.265	<0.0001	Yes
Clinical CDR	Vertical C/D Ratio	0.534	<0.001	0.339	0.0672	0.356	0.353	0.109	<0.001	Yes
Bulbus Length	Average RNFL Thickness	−0.534	<0.001	−0.302	0.1046	0.274	0.405	0.218	<0.01	No
Bulbus Length	RNFL Inferior Quadrant	−0.528	<0.001	−0.274	0.1425	0.238	0.380	0.218	<0.01	No
Bulbus Length	6 × 6 VD Parafoveal Superior	−0.555	<0.001	0.045	0.8130	<0.05	0.371	0.214	<0.01	No
Bulbus Length	ONH FI—Nasal	−0.611	<0.0001	−0.486	<0.01	0.495	0.512	0.237	<0.0001	Yes
Bulbus Length	ONH FI—Inferior	−0.567	<0.001	−0.368	<0.05	0.343	0.406	0.193	<0.001	Yes
Bulbus Length	ONH FI—Outer Region	−0.576	<0.001	−0.487	<0.01	0.639	0.454	0.214	<0.0001	Yes
Spherical Equivalent	ONH FI—Outer Region	0.540	<0.001	0.435	<0.05	0.375	0.392	0.376	0.686	No
Spherical Equivalent	ONH FI—Inferior	0.563	<0.001	0.207	0.280	0.053	0.397	0.376	0.611	No
**ONH Flow/Perfusion Index and RNFL Correlations (27 Correlations)**										
ONH FI—Outer Region	RNFL Superior Quadrant	0.599	<0.0001	0.580	<0.01	0.911	0.493	0.274	<0.001	Yes
ONH FI—Outer Region	RNFL Inferior Quadrant	0.590	<0.001	0.516	<0.01	0.682	0.425	0.274	<0.01	No
ONH FI—Outer Region	RNFL Nasal Quadrant	0.586	<0.001	0.345	0.072	0.238	0.453	0.274	<0.001	Yes
ONH FI—Outer Region	RNFL Hour 1	0.627	<0.0001	0.472	<0.05	0.396	0.490	0.274	<0.001	Yes
ONH FI—Outer Region	RNFL Hour 2	0.559	<0.001	0.010	0.960	<0.05	0.397	0.274	<0.05	No
ONH FI—Outer Region	RNFL Hour 3	0.543	<0.001	0.251	0.197	0.182	0.390	0.274	<0.05	No
ONH FI—Outer Region	RNFL Hour 7	0.549	<0.001	0.420	<0.05	0.521	0.431	0.274	<0.01	No
ONH FI—Nasal	RNFL Superior Quadrant	0.771	<0.0001	0.495	<0.01	0.070	0.635	0.315	<0.0001	Yes
ONH FI—Nasal	RNFL Inferior Quadrant	0.676	<0.0001	0.479	<0.01	0.254	0.515	0.315	<0.001	Yes
ONH FI—Nasal	RNFL Nasal Quadrant	0.694	<0.0001	0.344	0.068	0.059	0.492	0.315	<0.001	Yes
ONH FI—Nasal	RNFL Hour 1	0.717	<0.0001	0.471	<0.01	0.139	0.615	0.315	<0.0001	Yes
ONH FI—Nasal	RNFL Hour 2	0.660	<0.0001	−0.037	0.849	<0.01	0.435	0.315	<0.05	No
ONH FI—Nasal	RNFL Hour 3	0.577	<0.001	0.183	0.342	0.073	0.431	0.315	<0.05	No
ONH FI—Nasal	RNFL Hour 4	0.590	<0.001	0.422	<0.05	0.390	0.524	0.315	<0.001	Yes
ONH FI—Nasal	RNFL Hour 7	0.593	<0.001	0.294	0.122	0.151	0.496	0.315	<0.001	Yes
ONH FI—Nasal	RNFL Hour 11	0.536	<0.001	0.123	0.526	0.072	0.476	0.315	<0.01	No
ONH FI—Nasal	RNFL Hour 12	0.637	<0.0001	0.403	<0.05	0.218	0.541	0.315	<0.0001	Yes
ONH FI—Inferior	RNFL Hour 1	0.597	<0.001	0.387	<0.05	0.294	0.476	0.245	<0.001	Yes
ONH FI—Inferior	RNFL Hour 3	0.552	<0.001	0.287	0.132	0.223	0.401	0.245	<0.01	No
**Transition from Flow Index (FI) to Perfusion Flow (PF) Parameters**										
ONH PF—Outer Region	Average RNFL Thickness	0.551	<0.001	0.443	< 0.05	0.579	0.517	0.392	<0.01	No
ONH PF—Outer Region	RNFL Nasal Quadrant	0.643	<0.0001	0.217	0.259	<0.05	0.551	0.392	<0.001	Yes
ONH PF—Outer Region	RNFL Hour 2	0.646	<0.0001	0.092	0.636	<0.01	0.518	0.392	<0.01	No
ONH PF—Nasal	Average RNFL Thickness	0.610	<0.0001	0.130	0.502	<0.05	0.425	0.302	<0.05	No
ONH PF—Nasal	RNFL Nasal Quadrant	0.709	<0.0001	0.197	0.305	<0.01	0.515	0.302	<0.001	Yes
ONH PF—Nasal	RNFL Hour 2	0.748	<0.0001	0.036	0.853	<0.001	0.518	0.302	<0.001	Yes
ONH PF—Nasal	RNFL Hour 4	0.556	<0.001	0.128	0.507	0.059	0.458	0.302	<0.01	No
ONH PF—Superior	RNFL Hour 12	0.548	<0.001	0.576	<0.01	0.873	0.372	0.100	<0.001	Yes
**FAZ/Macular Correlations (3 Correlations)**										
FAZ Perimeter	3 × 3 PD central	−0.674	<0.0001	−0.372	<0.05	<0.05	0.447	0.039	<0.0001	Yes
FAZ Area	3 × 3 PD central	−0.708	<0.0001	−0.343	0.069	0.106	0.412	0.034	<0.0001	Yes
3 × 3 PD central	Macula Cube Total Thickness	0.648	<0.0001	0.445	<0.05	0.261	0.622	0.317	<0.0001	Yes

*p*-values are presented as follows: exact values with three decimal places for *p* > 0.05; otherwise reported as <0.05, <0.01, <0.001, or <0.0001. Statistically significant *p*-values (*p* < 0.001) in the univariable analysis are highlighted in yellow. Abbreviations: FAZ, foveal avascular zone; FI, flow index; GCIPL, ganglion cell–inner plexiform layer; MT, macular thickness; ONH, optic nerve head; PD, perfusion density; PF, perfusion; RNFL, retinal nerve fiber layer; VD, vessel density.

## Data Availability

The dataset contains sensitive patient information from pediatric participants. Despite anonymization, public deposition would potentially compromise patient privacy and violate the ethical approval conditions from the Ethics Committee of the Medical Association of Westfalen-Lippe (No. 2019-245-f-s) and institutional data-sharing policies. German data protection regulations (DSGVO/GDPR) additionally restrict public availability of medical data. Anonymized data and analysis scripts are available from the corresponding author upon reasonable request for legitimate research purposes and subject to appropriate data sharing agreements.

## Abbreviations

AL	Axial Length
BCVA	Best Corrected Visual Acuity
C/D	Cup-to-Disc Ratio
FAZ	Foveal Avascular Zone
FI	Flow Index
GCIPL	Ganglion Cell–Inner Plexiform Layer
GCL	Ganglion Cell Layer
ILM	Internal Limiting Membrane
IOP	Intraocular Pressure
IPL	Inner Plexiform Layer
LogMAR	Logarithm of Minimum Angle of Resolution
MT	Macular Thickness
OCT	Optical Coherence Tomography
OCT-A	Optical Coherence Tomography Angiography
ONH	Optic Nerve Head
PD	Perfusion Density
PF	Perfusion
R^2^	Coefficient of Determination
R^2^c	Coefficient of determination of the confounder-only model
RGC	Retinal Ganglion Cells
RNFL	Retinal Nerve Fiber Layer
RPC	Radial Peripapillary Capillaries
RPE	Retinal Pigment Epithelium
RT	Retinal Thickness
SD	Standard Deviation
VD	Vessel Density
